# Could Kallikrein-Related Serine Peptidase 3 Be an Early Biomarker of Environmental Exposure in Young Women?

**DOI:** 10.3390/ijerph18168833

**Published:** 2021-08-21

**Authors:** Salvatore Raimondo, Mariacira Gentile, Giusy Esposito, Tommaso Gentile, Ida Ferrara, Claudia Crescenzo, Mariangela Palmieri, Felice Cuomo, Stefania De Filippo, Gennaro Lettieri, Marina Piscopo, Luigi Montano

**Affiliations:** 1 “Gentile s.a.s.” Research Center, 80054 Gragnano, Italy; salvatoreraimondo57@gmail.com (S.R.); mariaciragentile@gmail.com (M.G.); giusy.esposito890@libero.it (G.E.); labgentile@virgilio.it (T.G.); cmuflc@gmail.com (F.C.); dfstefy74@gmail.com (S.D.F.); 2 Medical Center “Gunè”, 80011 Acerra, Italy; idaferrara@libero.it; 3 Clinic Center “HERA”, 80014 Giugliano, Italy; claudia.crescenzo@gmail.com (C.C.); m.palmieri@clinicahera.it (M.P.); 4 Department of Biology, University of Naples Federico II, 80126 Naples, Italy; gennarole@outlook.com; 5 Andrology Unit and Service of Lifestyle Medicine in UroAndrology, Local Health Authority (ASL) Salerno, Coordination Unit of the network for Environmental and Reproductive Health (Eco-FoodFertility Project), “Ospedale San Francesco D’Assisi”, Oliveto Citra, 84020 Salerni, Italy; 6 PhD Program in Evolutionary Biology and Ecology, University of Rome “Tor Vergata”, 00133 Rome, Italy

**Keywords:** KLK3, pollution, menstrual cycle, environmental marker, reproductive health, environmental exposure, Land of Fires, EcoFoodFertility, progesterone

## Abstract

Bisphenols and phthalates affect androgen receptor-mediated signaling that directly regulates Kallikrein-Related serine Peptidase 3 (KLK3) secretion, indicating that environmental factors may play a role in KLK3 secretion. With the aim of obtaining preliminary data on whether KLK3 could serve as an early marker of environmental pollution effects, in 61 and 58 healthy women living in a high environmental impact (HEI) and low environmental impact (LEI) area, respectively, serum KLK3 levels at different phases of menstrual cycle were measured. KLK3 values resulted in always being higher in the HEI group with respect to the LEI group. These differences were particularly relevant in the ovulatory phase (cycle day 12°–13°) of the menstrual cycle. The differences in KLK3 values during the three phases of the menstrual cycle were significant in the LEI group differently from the HEI group. In addition, higher progesterone levels were observed in the LEI group with respect to the HEI group in the luteal phase, indicating an opposite trend of KLK3 and progesterone in this phase of the menstrual cycle. Although changes in KLK3 could also depend on other factors, these preliminary data could be an early indication of an expanding study of the role of biomarkers in assessing early environmental effects for female reproductive health.

## 1. Introduction

Kallikreins are a subgroup of serine proteases responsible for various physiological functions and are capable of cleaving peptide bonds in proteins. In humans, plasma kallikrein (KLKB1) has no known homologs, whereas tissue peptidase-related kallikrein (KLKs) constitute a family of 15 highly conserved serine proteases. These genes are localized on chromosome 19q13, forming the largest contiguous group of proteases in the human genome. The 15 members of the kallikrein-related serine peptidase (KLK) have different tissue-specific expression profiles. Initially, KLK3 was believed to be absent from female tissues and fluids. However, KLK3 has been detected in some female tissues (including breast, ovarian, and endometrial tissues) and body fluids (serum, breast milk, amniotic fluid). The presence of KLK3 in these female tissues appears to be closely associated with the regulation of steroid hormones, particularly androgens and progestins. Estrogens themselves do not appear to affect KLK3 regulation, but they may impair androgen-induced KLK3 production. In tissues and fluids, KLK3 can be found in two molecular forms: free KLK3, which represents the enzymatically active form, and in complexed form when bound to protease inhibitors. Recent studies [1,2,3] suggest that KLK3 can no longer be considered only as a prostate-specific marker, but as a protein that could be produced under conditions of steroid hormone stimulation. Kallikreins are a rich source of disease biomarkers. The kallikrein locus is extraordinarily responsive to steroids and other hormones; in fact, at least 14 functional hormone response elements have been identified. The highest detectable proportion of the KLK3 protein in serum is secreted by the prostate gland, and considerably higher concentrations, approximately 20,000 fold higher, can also be detected in human seminal plasma [4]. KLK3 has been detected in salivary glands, brain, breast, and other tissues, although at concentrations 100 times lower than the serum level [5]. Recent studies [6] have reported the presence of KLK3 in female serum in relation to polycystic ovary syndrome (PCOS) [7] and hirsutism [8,9]. Of particular interest is vaginal cervical fluid (CVF), which is a complex biological fluid that hydrates the mucosa of the lower female reproductive system. Extensive proteomic and biochemical studies on CVF have revealed that it contains large amounts of endogenous proteases and protease inhibitors including an abundance of several members of the tissue kallikrein-related peptidase (KLK) family. The roles of KLKs in the lower female reproductive system are not fully understood, their activation is pH-dependent, and there are also various other modes of regulation in the vagina. Roles have been postulated in physiological related to antimicrobial processes, vaginal and cervical epithelial desquamation, sperm transport, and fetal membrane processing, as observed in premature membrane rupture, through the binding of known and unknown kallikrein substrates [10]. Despite their presence in human tissues and fluids, expression levels of KLKs vary widely, with the highest expression observed in reproductive-related tissues and fluids. Zarghami and colleagues [11] reported that KLK3 is secreted cyclically during the menstrual cycle and appears to follow the peak concentration of progesterone, with a delay of 10–12 days. Serum KLK3 concentrations are highest during the mid-to-late follicular phase, fall continuously with a half-life of 3–5 days between the late follicular phase and mid-cycle, and reach a minimum during the mid-luteal phase. In many tissues, kallikrein expression is regulated by steroid hormones. In fact, the kallikrein locus is exceptionally responsive to hormones because each kallikrein is upregulated by multiple hormones. For this reason, many researchers use kallikreins as a marker of hormone receptor activity [1,12]. Progesterone regulates kallikrein expression in various tissues, sometimes through direct binding of the PR (progesterone receptor) to kallikrein promoters. KLK2 and KLK3 are both upregulated by progesterone in breast cancer cell lines [13]. KLK3 is directly related to androgen and estrogen receptor upregulation. Several data suggest that various kallikreins are regulated by steroid hormones; in particular, androgens stimulate the KLK3-secreting cell line while estrogens stimulate the KLK10, KLK11, and KLK14-secreting cell line. Furthermore, KLK3 expression is downregulated by combined stimulation, confirming that estrogens can antagonize and block androgen receptor activity [14]. Therefore, acquiring more detailed information about the mechanisms regulating kallikrein expression should be a priority and the kallikrein locus could be an important model in the era of genome-wide analyses. In particular, a more complete understanding of the transcriptional regulation of kallikreins may help to formulate more concrete hypotheses about the physiological functions of kallikreins and their potential usefulness as biomarkers. For example, there is evidence that plasma prekallikrein (pKal), together with coagulation factor XII (FXII) and high molecular weight kininogen (HK), form the plasma kallikrein–kinin system (KKS), a component of the innate immune response that generates proinflammatory products in response to injury [15]. Furthermore, using genetically modified animal models, Wang et al. (2019) demonstrated the critical role that plasma kallikrein plays in PM2.5-induced lung injury, revealing a novel function of this system [16] and also suggested that the strategy targeting this factor could have protective effects [16]. In the present work, the choice of KLK3 as a possible new early biomarker of environmental exposure in young women was based on evidence reporting the possibility that some environmental pollutants could activate KLK3 secretion by prostatic epithelial cells in vitro [17]. In addition, other chemicals such as bisphenols, phthalate/DBP (dibutyl phthalate) affect AR (androgen receptors) mediated signaling that directly regulates KLK3 secretion. This could suggest that environmental factors may play a role in KLK3 secretion [18]. Based on these observations, in the present work, we studied the serum concentration levels of KLK3 during the menstrual cycle in women from areas with different environmental impact. The purpose of this study is to provide preliminary data on the possible role of KLK3 as a new biomarker to assess the early effects of environmental exposure for female reproductive health. With this work, we also want to arouse the scientific curiosity of other scientists in other areas to investigate and compare our data so that our idea of environmental investigation can be supported.

## 2. Materials and Methods

### 2.1. Ethical Statements

This study was made within the EcoFoodFertility project (available online http://www.ecofoodfertility.it (accessed on 30 July 2015). All methods were carried out in accordance with the Code of Ethics of the World Medical Association (Declaration of Helsinki) guidelines and regulations. All experimental protocols were approved by the Ethical Committee of the Local Health Authority Campania Sud-Salerno (Committee code 43/2015/06). Informed consent was obtained from all recruited subjects.

### 2.2. Study Areas and Recruitment

**Figure 1:**
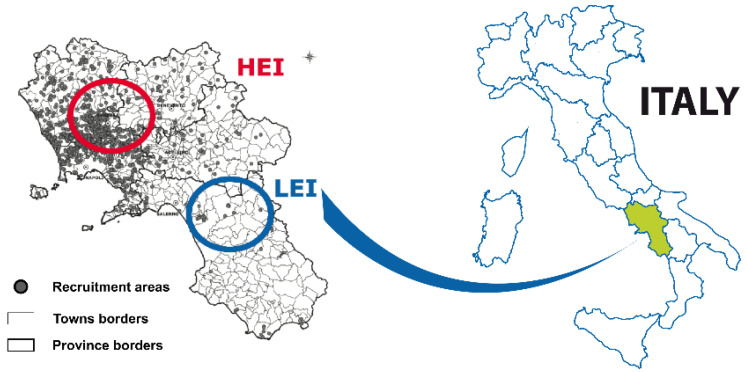
The figure shows the two study areas. The high environmental impact (HEI) area is highlighted in red and the low environmental impact (LEI) area is highlighted in blue.

A total of 119 women were enrolled in this study from September 2017 to April 2020 within the EcoFoodFertility project (www.ecofoodfertility.it accessed on 23 June 2018), a multidisciplinary study connecting human lifestyle and dietary habits to the environmental consequences of exposure to toxicants. Of these 119 women, 61 participants, aged 28.66 ± 4.43, lived in an area of the Campania region (southern Italy) with high environmental impact (HEI) (DL 136/2013-l 6/2/2014, ARPAC—Regional Environmental Protection Agency of Campania), the so-called “Land of Fires” for the multiplicity of sources of pollution (illegal disposal of urban, toxic and industrial wastes, dumping practices, traffic, intensive agriculture) [18,19,20,21,22,23,24]. The remaining 58 participants, aged 27.3 ± 3.18, lived in an area of the same region but with low environmental impact (LEI) (Figure 1). Signed informed consent was received from all participants in this study, in accordance with the ethical principles of experimentation (institutional or regional) and the Declaration of Helsinki of 1975, revised in 2000. None of the participants works in a job that poses a health risk or suffers from chronic diseases. In Table 1 are shown the jobs of the participants of the study.

The blood samples of the first group (*n* = 58 healthy females) were obtained from San Francesco d’Assisi Hospital in Oliveto Citra Province of Salerno. This is a municipality belonging to the low environmental impact area (LEI) called “Alto-medio Sele” (Oliveto Citra, Contursi Terme, San Gregorio Magno, Buccino, Ricigliano, Valva and Colliano). This area is considered a low environmental impact by the regional authority of environment (ARPAC. (Available online: http://www.arpacampania.it/aria (accessed on 3 August 2017). The economy of this area is principally based on low to medium scale agriculture with no known illegal disposal of toxic waste (blue circle in Figure 1). Blood samples from the second group (*n* = 61 healthy females) were obtained from municipalities belonging to the ‘Land of fires’ (Acerra, Caivano, Afragola, Casalnuovo, Pomigliano d’Arco, Brusciano, Giugliano, Cardito, and Marigliano) and the center of Medicina Futura (Acerra-Province of Naples) (red circle in Figure 1). “Land of Fires” is an area of high environmental impact (HEI) in Campania that is socially recognized, on the basis of the report of the Campania Region Environmental Protection Agency, as an area with the highest concentration of illegal toxic waste disposal sites. The contamination result of unlawful garbage dumping from civil, industrial, and hospital operations. Waste from these activities has been illegally deposited and burned in this location, resulting in harmful contamination of nearby agricultural lands and aquifers [25] (ARPAC. Available online: http://www.arpacampania.it/aria (accessed on 3 August 2017). In this area, numerous studies indicate a high incidence of chronic degenerative diseases as a consequence of pollution [19,20,21,26].

A specific “participant form” was proposed in which anamnestic and clinical data are reported in order to create a database. Participants reported symptoms and signs of any medical conditions, hirsutism, acne, and anamnestic data related to their lifestyle, use and/or abuse of alcohol, smoking, and possible drug use. Participants had no major chronic diseases and had resided permanently in the selected areas for at least five years, were not professionally exposed to risk factors, and had not taken oral contraceptive pills for at least two years. They had not used drugs in the 12 months before the blood draw. Participants were asked about their age at menarche, whether they were nulliparous/multiparous, and whether they had experienced spontaneous/voluntary abortions. The participants had normal menstruation, the duration of the whole cycle varied from 28 to 30 days; and regularity, volume, and duration were regular. In addition, BMI (body mass index), waist circumference, waist-to-hip ratio, and Ferriman–Gallwey score for hirsutism were calculated.

### 2.3. Blood Sampling and Determination of KCL3

Blood samples were collected during the three phases of the menstrual cycle, namely on days 5°–6° (follicular phase), 12°–13° (ovulatory phase), and 19°–20° (luteal phase). KLK3 was assayed in all three sera samples and progesterone was assayed on the third samples.

The method used for the KLK3 assay in serum was the Access Hybritech KLK3 assay, developed by Beckman Coulter (Brea, CA, USA), which is a two-site immunoenzymatic assay, also known as a sandwich immunoassay, using the principle of chemiluminescence and produces light directly proportional to the KLK3 concentration in the sample. 

### 2.4. Determination of Progesterone Levels

Progesterone levels were determined using the Access 2 Immunoassay System by Beckman Coulter according to the manufacturer’s instructions. 

### 2.5. Statistical Analyses

The whole database, covering all participants, was subjected to a statistical analysis for each area and for the entire enrolled population. Assuming a significant difference between the groups belonging to the two selected areas, differences were found between the 61 participants from the high environmental impact area and the 58 participants from the low environmental impact area. A statistical analysis for each area and for the whole enrolled population was performed across the two groups using the one-way ANOVA test followed by Bonferroni adjustment for multiple comparisons. The statistical analyses were performed by GraphPad Prism 9 (ver. 9.1.2 (226))( GraphPad Software, San Diego, CA, USA).

## 3. Results

### 3.1. Analysis of the Characteristics of Participants Residing in HEI and LEI Areas

Table 2 shows the variables of the two groups under examination; data were obtained from the forms completed by the participants and from our clinical evaluations. KLK3 concentrations in the serum of the young women showed an irregular behavior. Indeed, in 14 out of 58 participants belonging to the low-impact group (24.1%), and in 17 out of 61 participants belonging to the high-impact group (27.8%), KLK3 was not detectable in any of the three samples per menstrual cycle with the method we used (detection limit 0.001 pg/mL). Therefore, only 88 out of all participants (73.9%) were included in the further statistical analysis and comparative evaluation (Table 2). The jobs of the excluded participants were included in those already listed. The BMI of the two groups was homogeneous. In addition, Ferriman–Gallwey score, which is used to evaluate hirsutism, was compared with KLK3. The Ferriman–Gallwey score [27], is widely used to assess and quantify hirsutism in women. The method evaluates nine regions of the body (excluding legs and forearms) for hair growth, with the score ranging from 0 (no visible hair growth) to 4 (extensive visible hair growth) in each of the nine areas. The score can range from 0 to 36: score 1 = score 1–9 (normal); score 2 = score 10–18 (mild Hirsutism); score 3 = score 19–27 (moderate Hirsutism); score 4 = score 28–36 (severe Hirsutism). In this study, the participants belonged only to scores 1 and 2.

### 3.2. Analysis of KLK3 in the Three Phases of Menstrual Cycle

**Figure 2:**
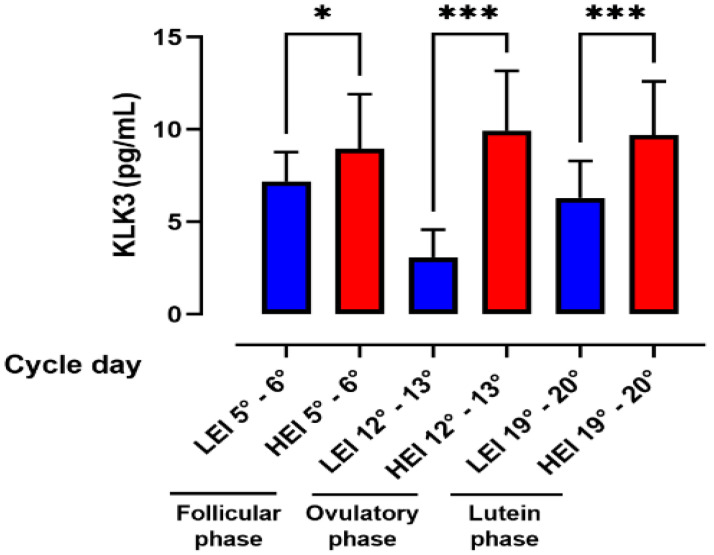
KLK3 values during the three phases of menstrual cycle in LEI (blue) and HEI (red) groups. Values are presented as mean ± S.D. Asterisks indicate a statistically significant difference between the two groups: * = *p* < 0.05; *** = *p* < 0.001.

Figure 2 shows the distribution of KLK3 levels in the three phases of the menstrual cycle in the LEI and HEI groups. Changes in KLK3 values were statistically significant in the LEI group. In fact, comparison of values between the follicular phase (7.18 ± 1.57 pg/mL) and the ovulatory phase (3.07 ± 1.49 pg/mL) showed a significant decrease while the comparison of values between the ovulatory phase (3.07 ± 1.49 pg/mL) and the luteal phase (6.29 ± 1.97 pg/mL) showed a significant increase.

In contrast, the results obtained on the HEI groups showed that the highest concentrations of KLK3 were found on days 12–13 of the menstrual cycle (9.932 ± 3.240 pg/mL). Changes in KLK3 values in the three cycle phases of follicular phase (8.968 ± 2.940), ovulatory phase 9.932 ± 3.240 and luteal phase (9.702 ± 2.905) were not statistically significant.

Moreover, in all phases, the HEI group showed significant higher levels of KLK3 in comparison with the LEI group. These variations were particularly evident during the ovulatory phase of the cycle (days 12°–13°). In addition to differing significantly only in the LEI group in the three phases of the menstrual cycle, it is interesting to note that KLK3 values in the different phases of the menstrual cycle followed the opposite trend in the LEI and HEI groups. In particular, the LEI group had similar KLK3 values in the follicular and luteal phases, while there was a drastic reduction in KLK3 levels in the ovulatory phase. In contrast, the HEI group had similar values in the follicular phase and luteal phase, but a slight increase in the ovulatory phase (Figure 2). 

### 3.3. Analysis of the Correlation between KLK3 Values in Nulliparous and Multiparous

**Figure 3:**
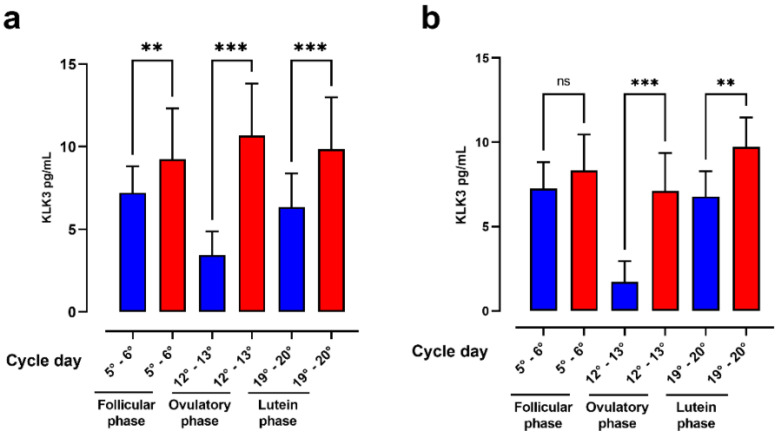
The figure shows the bar plot of nulliparous LEI vs. HEI (**a**) and multiparous LEI vs. HEI (**b**). LEI = blue, HEI = red. Asterisks indicate a statistically significant difference between the two groups. The data show as mean ± S.D. ns = not significance; ** = *p* < 0.01; *** = *p* < 0.001.

**Figure 4:**
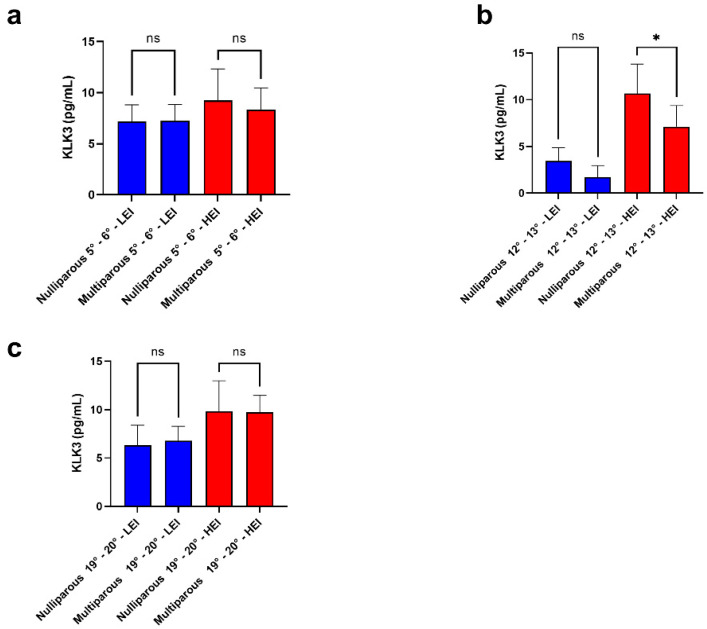
The figure shows the comparison between nulliparous and multiparous in the three groups of cycle days. Subfigure (**a**) (5°–6°), (**b**) (12°–13°) and (**c**) (19°–20°) cycle days, respectively. LEI: low environmental impact area; HEI: high environmental impact area. ns = not significance; * = *p* < 0.05.

The correlation analysis between KLK3 values in nulliparous and multiparous women, illustrated in Figure 3, showed that in the nulliparous, the KLK3 value was always higher in the HEI group respect to LEI group. In the latter, there was a significant decrease in the KLK3 value in the ovulatory phase. Additionally, in the case of multiparous, the KLK3 value was higher in comparison with the LEI group and once again, the major KLK3 value was observed in the ovulatory phase. Figure 4 shows the comparison between nulliparous and multiparous in the three groups of cycle days.

### 3.4. Analysis of Progesterone Levels

**Figure 5:**
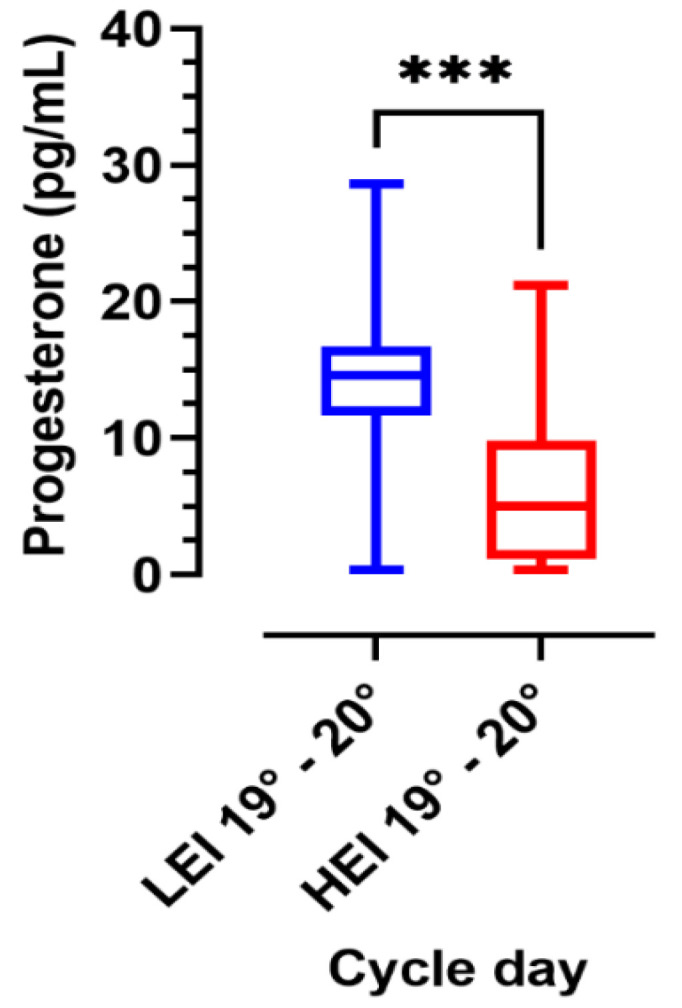
Box and plots of progesterone values in the two groups (LEI: blue; HEI: red) (LEI *n* = 44; HEI *n* = 44). Asterisks indicate a statistically significant difference between the two groups: *** = *p* < 0.001.

The analysis of progesterone levels in the luteal phase (cycle days 19°–20°) in the two groups, is shown in Figure 5. Significant differences were found between the two groups with higher values in the LEI group than in the HEI group. 

## 4. Discussion

It is well known that environmental pollution has negative consequences on the health of various organisms including humans, and also on their reproduction [28,29,30,31,32,33,34,35,36,37]. Very few studies have been conducted on the impact of pollution on the female reproductive system versus the male one. This is because it is easier to collect and study male gametes. Both animal and human epidemiological studies support the idea that air pollutants cause defects during gametogenesis, leading to a drop in reproductive capacities in exposed populations [38]. In this context, the development of studies exploiting biomarkers of exposure together with effective biomarkers is necessary. Numerous biomarker studies have already been carried out in the Land of Fires to identify pollution-related changes in this area [19,20,24,39]. “Land of Fires” is known as an area of high pollution and is recognized by the regional environmental agency and the Ministry of Environment and Health. There are numerous publications that indicate this area with a high incidence of chronic-degenerative diseases and high rates of pollutants. In addition to the studies that confirm this area as polluted and has health effects evaluated epidemiologically, our data and publications, which were conducted with a biomonitoring system between the two areas on semen as an early indicator and discriminates even better than blood of the effects of environmental exposure, suggest that our attempt to also assess biomarkers of exposure on the female side could be an important approach for primary prevention. For these reasons, in order to identify a biomarker of exposure typical of women, in the present work, we analyzed women residing in low and high environmental impact areas to assess serum KLK3 levels at different phases of the menstrual cycle by using an ultra-sensitive kit for KLK3 with a detection limit of 0.001 ng/mL. This work is an original study, as in the literature, there is no scientific evidence of correlation between the secretion of KLK3 and environmental impact. The idea comes from a study of human prostatic epithelial cells in vitro that, when put in contact with pollutants, were able to activate the secretion of PSA. Skene’s glands, secretors of KLK3 in women, are reached by blood by endocrine-disrupting chemicals (EDC) present in the blood circulation, and this way is the focus of our research. In addition, that kallikrein can be affected by environmental pollutants was reported in several papers as early as 1993 when were demonstrated alterations in kallikrein activity in the urine of men after chronic exposure to mercury, lead, and cadmium, indicative of a metabolic disorder or dysfunction of the distal tubule [40,41,42].

In this work, the number of participants of LEI was 58 and HEI was 61, however, the total number in Table 1 do not match these numbers because only 88 out of all participants were included in the further statistical analysis and comparative evaluation, since only 75.9% of the tested women showed values of KLK3 greater than 0.001 ng/mL, while in 24.1% of the participants, the KLK3 was not detectable. For these cases, we can hypothesize a lack of secretion of KLK3 or a low secretion not detectable by the method we used, and for this reason, these women were excluded from the statistical evaluation. However, it should be noted that the percentage of non-dosability occurred more in LEI participants, and, therefore, raised the consideration that the organism has the ability to respond to external stimuli in different ways.

Due to this diversity of response to the KLK3 dosage, a sort of “resistance” or “sensitivity” to toxic substances can be hypothesized, perhaps due to the duration of exposure or the type of exposure. An example of this is the behavior of cadmium, which, unlike other toxic substances, has the ability to diffuse easily in the organism due to its properties to penetrate the barriers that separate the bloodstream from the tissues and diffuse in the biological fluids of the organism [43]. Previous studies have reported detectable KLK3 in 50% of non-pregnant women [39,40,41,42,43,44,45]. In 1996, Filella and colleagues [46] detected the presence of KLK3 in 58% of female sera. Escobar-Morreale and colleagues [47] in 1998 detected the presence of 36.4% KLK3 in the serum of women with normal menstrual cycles and Mannello and colleagues [48] in 2001 did not detect the presence of KLK3 in female serum. Normal serum levels of KLK3 in women range from 0.02–0.06 ng/mL [49]. In our study, in the participants belonging to the LEI group, high levels of KLK3 were found during days 5–6 of the menstrual cycle (7.183 ± 1.593) and a second, smaller peak was observed on days 19–20 (6.298 ± 1.996). Su and colleagues found low levels of KLK3 during days 10–23 and a peak at the end of the cycle or at the beginning of the next cycle [50]. In our study, instead, as already mentioned, for the LEI group, the KLK3 peak was observed both at the beginning and at the end of the menstrual cycle.

In the LEI group, KLK3 values significantly decreased between the follicular and ovulatory phase and then increased in the luteal phase, while in the HEI group, the changes in KLK3 values in the three cycle phases were not statistically significant. 

In this work, we also evaluated the progesterone levels on days 19–20 of the cycle in the LEI and HEI groups. Black and colleagues in 1972 [51] found mean progesterone levels of 1.8 ± 1.1 pg/mL (days 0–11 of the cycle), 3.1 ± 1.8 (days 12–14 of the cycle), 12.1 ± 7.8 pg/mL (days 15–25 of the cycle), and 4.3 ± 3.3 pg/mL (days 26–28 of the cycle). In our study, progesterone values were measured on days 19–20 of the cycle. The mean value obtained in the LEI group was 14.1 ± 6.19 pg/mL while in the HEI group, the value was 6.67 ± 5.73 ng/mL. We found statistically significant (*p* < 0.0001) changes in progesterone values among the two groups studied, depending on their residence. Progesterone is the main endocrine product of the corpus luteum during the menstrual cycle [11]. Corpus luteum insufficiency is not predictable and ovulation is not a certain event even for normomestruate women. The two groups of participants examined were all normomestruate at the time of acceptance to participate in the project. The assessment of progesterone is only indicative of how KLK3 behaves in the three phases of the cycle in relation to ovulation, taking into account that progesterone is the only way to understand if a menstrual cycle is ovulatory or not.

In vitro studies have shown that sera obtained during the menstrual cycle can stimulate KLK3 production in a breast cancer cell line (T-Line 47D) [11]. Measurement of KLK3 protein and KLK3 mRNA levels showed that the ability of serum to induce KLK3 production in cancer cells was proportional to serum progesterone levels, with the greatest stimulation occurring with serum containing more progesterone (days 22–24 of the menstrual cycle). In addition, with luteal phase serum, KLK3 mRNA expression was significantly increased. 

In 75.9% of participants in LEI and 83.6% of participants in HEI, KLK3 was detectable in serum and was expressed in the three phases of the menstrual cycle: in the follicular phase, the minimum value was 3.52 pg/mL and the maximum 17.74 pg/mL; in the ovulatory phase, the minimum value was 0.11 pg/mL and the maximum value was 20.36 pg/mL; and in the luteal phase, the minimum value was 1.12 pg/mL and the maximum value was 19.24 pg/mL. Therefore, the concentration of KLK3 in the blood serum of the young women showed a significant increase in the ovulatory phase of the HEI group with respect to the LEI group. Only the nulliparous 12°–13° HEI vs. multiparous 12°–13° HEI groups showed a significant difference. Although these data may indicate a different trend between nulliparous and multiparous women, the different number of multiparous and nulliparous women examined in this study does not allow for a conclusion on this aspect. Therefore, an attempt will be made in the future to examine a greater number of women of the two types in order to have significant results. This also because a study performed in China in 2020, which demonstrated that exposure to PM2.5 during the first trimester tended to be associated with an increased risk of hypertensive disorders of pregnancy (HDP). The effect estimates are more obvious for nulliparous women than multiparous women [52].

As previously reported, comparison of KLK3 values with the anamnestic data showed a significant change (*p* < 0.05) between the two groups of participants for alcohol use and previous abortions; semeiotic evaluations between the two groups showed a significant change (*p* < 0.05) for the waist/hip ratio. Other works have reported a direct causal link between environmental impact and some clinical manifestations attributable to EDC substances. In the two groups of participants examined, we reported the data of these clinical manifestations (BMI, hirsutism, and waist/hip circumference). Changes in KLK3 concentrations have been reported in several studies reflecting serum progesterone levels, albeit with a delay of 12 to 16 days. In a biphasic profile, KLK3 is produced in target tissues in response to increased progesterone levels in the ovulatory phase, KLK3 diffuses into the blood and is detected in the luteal phase. Progesterone spikes during the luteal phase are able to provide a spike in KLK3 in the first follicular phase of the next cycle [6,11]. The interpretation of the negative and positive peaks of KLK3 found in the serum of LEI and HEI area participants, respectively, is different; most probably a mechanism of KLK3 secretion is independent of progesterone. In the HEI area participants, this value was found to be higher on average with no significant variation between the three phases of the menstrual cycle. This result could suggest that other stimuli such as environmental factors prompt a continuous secretion by the epithelial cells of the glands by Skene. Indeed, in other studies, it has been shown that prostate epithelial cells respond to the stimulation of certain pollutants [17]. In addition, our previous studies performed on the spermatozoa of subjects domiciled in the same areas reported that there was significant sperm DNA damage in HEI areas [29,31,34]. We can hypothesize that in HEI areas, there are environmental pollutants that may be diffused in the area and/or in the soil (food or water) that may interfere with secretory tissues including Skene’s glands. Of course, future studies will lead us to better define the responsible pollutants.

After all, it is well known that air pollution can affect women’s reproductive health, specifically menstrual cycle characteristics, oocyte quality, and risk of miscarriage. In particular, it is also reported that luteal phase shortening, a possible manifestation of luteal phase deficiency, can be affected by air pollution, specifically by the toxicants released by fossil fuel combustion [53]. The concentrations of KLK3 in the blood serum of LEI area participants showed a negative peak in the ovulatory phase with significant changes (*p* < 0.0001) between the follicular and ovulatory phase and the ovulatory and luteal phase, and this cyclicality seems to respond to endogenous stimuli.

Furthermore, comparing the data of the two groups, we noted a significantly different (*p* < 0.001) dosage rate in the samples of the three cycle phases: 75.9% for the LEI and 83.6% for the HEI area, respectively. This may be the result of increased stimulation that may occur in the HEI area. The roles of KLK3 in the female reproductive system are not fully known. The activation of KLK3 in vaginal cervical fluid (CVF) is dependent on vaginal pH, but there are other mechanisms that regulate KLK3 in the vagina. It has been hypothesized that KLK3 plays a role in physiological functions related to antimicrobial processes, vaginal and cervical epithelium desquamation, spermatozoa transport and movement, and more recent works have indicated a probable role as an immunoregulator. All this, also considering that environmental pollution may impact pregnancy outcomes, was reported by Wojtyla et al. (2020). In fact, these authors demonstrated that environmental pollution produced lower birth weight, higher risk of giving birth to a child weighing <2500 g, and also to a child in a worse general condition, defined as APGAR scores [54]. In addition, it is well known that some pollutants can also affect the FSH:LH ratio. In fact, a subgroup of predominantly volatile, non-persistent PCBs increased the probability of having a higher FSH:LH ratio regardless of age in women with known exposure to PCBs and other persistent organic pollutants [55]. Several studies have suggested that the key to using kallikreins as biomarkers is to test them in combination; in fact, for colorectal, ovarian, and non-small cell lung cancer, kallikreins are more effective as multiparameter panels of biomarkers than as individual antigens. More detailed information on the transcriptional regulation of kallikreins would explain why they are dysregulated in particular diseases (prostate cancer) and, therefore, could represent biological variations as biomarkers [56,57,58,59,60]. Therefore, the observed changes of KLK3 in the participants of the two areas examined could represent a very first indication of an expanding study on the role of biomarkers as a promising way to assess the early effects of pollutants on female reproductive health. Moreover, on the male front, the human semen seems to represent an excellent biomarker of environmental exposure, thanks to the biomonitoring study we are conducting through the EcoFoodFertility project between the two areas [61,62]. Finally, focusing our attention on assessing biomarkers of exposure also on the female side can be an important approach for primary prevention and open new knowledge scenarios in the evaluation of the environment–health relationship. Of course, changes in KLK3 could also depend on other factors, therefore, further studies, larger numbers, and more homogeneous age group sampling will be needed to better understand the functions of KLK3 in a woman’s serum on different days of the cycle

## 5. Conclusions

This study provides preliminary indications on whether KLK3 could serve as an early marker of environmental pollution in cycling women. Environmental pollutants in the HEI area may exert an endocrine interference that stimulates the Skene’s glands; in fact, during the menstrual cycle, the concentrations of KLK3 are higher and with minimal fluctuations. The changes during the phases of the menstrual cycle demonstrate a different action other than simple secretion. The data obtained seem to go beyond the functions advanced by other authors on its role in antimicrobial processes, vaginal and cervical epithelial desquamation, and spermatozoa transport. The possible role of KLK3 as a biomarker of environmental exposure in women must obviously be validated over time with more consistent sample numbers and additional data. Ultimately, our work is intended to open up this scenario of an expanding study.

## Figures and Tables

**Table 1:** The table shows the jobs of the participants in the study.

Jobs	LEI	HEI
Shop assistants in clothing and accessories shops	12	10
Shop assistants in food shops	9	3
Students	14	16
Seasonal agricultural workers not in greenhouses	2	6
Secretaries in professional offices	4	2
Not studying and not working	3	7

**Table 2:** Variables of the two groups examined: High environmental impact (HEI) and low environmental impact (LEI). The data were obtained from questionnaires completed by the participants and from our clinical evaluations. ns = not significant.

Criteria	HEI Group (*n* = 44)	LEI Group (*n* = 44)	*p*-Value
Age (years.)	28.66 ± 4.43	27.3 ± 3.18	ns
Smokers	8.19%	5.17%	ns
Alcohol	9.84%	1.72%	ns
Drugs	not used-	not used	not applicable
Age of menarche (years)	11–13	11–12	ns
Nulliparous	86.9%	79.3%	ns
Multiparous	13.1%	20.7%	
Previous abortions	14.8%	3.4%	*p* < 0.05
BMI score	24.2–29.4	23.1–27.4	ns
Waist circumference (cm)	72–110	69–90	ns
Waist-to-hip ratio	0.81–0.95	0.55–0.78	*p* < 0.05
Ferriman–Gallwey score 1	75.4%	75.9%	ns
Ferriman–Gallwey score 2	24.6%	24.1%	ns

The numbers represent average ± SD, min to max or percentage.

## Data Availability

Data are within EcoFoodFertility research project.
